# Comparison of obstetric and perinatal complications in intracytoplasmic sperm injection cycles with autologous oocytes and donated oocytes

**DOI:** 10.1590/1806-9282.20240357

**Published:** 2024-08-16

**Authors:** Valéria Cristina Datrino Horta, Renato Augusto Moreira de Sá, Marco Antônio Pessanha Lourenço, Raphael Datrino Horta, Rodrigo Datrino Horta, Luiz Guilherme Louzada Maldonado, Alberto Borges Peixoto, Edward Araujo

**Affiliations:** 1 Fluminense Federal University, Department of Obstetrics – Niteroi (RJ), Brazil.; 2 Profertil Reproductive Medicine – Niteroi (RJ), Brazil.; 3 Bonsucesso Federal Hospital, Service of Gynecology and Obstetrics – Rio de Janeiro (RJ), Brazil.; 4 University Center of Valença, Department of Gynecology and Obstetrics – Valença (RJ), Brazil.; 5 Fertility Medical Group – São Paulo (SP), Brazil.; 6 University of Uberaba, Mario Palmério University Hospital, Gynecology and Obstetrics Service – Uberaba (MG), Brazil.; 7 Federal University of Triângulo Mineiro, Department of Obstetrics and Gynecology – Uberaba (MG), Brazil.; 8 Federal University of São Paulo, Paulista School of Medicine, Department of Obstetrics – São Paulo (SP), Brazil.; 9 Municipal University of São Caetano do Sul, Discipline of Woman Health – São Caetano do Sul (SP), Brazil.

**Keywords:** ICSI, Oocyte donation, Pregnancy outcomes, Neonatal intensive care units

## Abstract

**OBJECTIVE:**

The aim of this study was to compare the obstetric and perinatal complications in women who became pregnant with autologous oocytes and those who received donated oocytes (DO) in intracytoplasmic sperm injection cycles (ICSI).

**METHODS:**

A retrospective cohort study was carried out by collecting data from medical records between 2019 and 2022. Only patients who underwent ICSI in an induced cycle using their own or freshly DO, with male infertility factor and tubal factor, were included.

**RESULTS:**

A total of 120 patients were assessed, comprising 51 cases utilizing their own oocytes (control group) and 69 cases employing DO (study group). Patients receiving DO (n=69) exhibited a significantly higher mean age compared to those utilizing their own oocytes (n=51) (41.96±2.16 vs 38.54±1.42 years, p<0.001). There was no significant association between the source of oocytes and gestational age at delivery (p=0.296), birth weight (p=0.836), admission to neonatal intensive care unit (ICU) (p=0.120), or maternal admission to adult ICU (p=0.767). Additionally, the origin of oocytes did not demonstrate any significant association with the risk of pre-eclampsia (p=0.357), gestational diabetes mellitus (p=0.187), premature rupture of membranes (p=0.996), uterine atony (p=0.996), placenta previa (p=0.393), oligohydramnios (p=0.393), or gestational hypertension (p=0.393)."

**CONCLUSION:**

An increase in obstetric and perinatal complications was not observed in pregnancies with DO compared to pregnancies with autologous oocytes in women undergoing ICSI without prior comorbidities. Further studies with larger sample sizes are required to validate our findings.

## INTRODUCTION

According to the World Health Organization (WHO), infertility is defined as the failure of a couple to achieve pregnancy after 1 year of consistent, unprotected sexual intercourse. Globally, infertility impacts around 17.5% of couples in their reproductive years, as reported by the WHO. It is noteworthy that infertility can stem from factors originating from both males and females^1–3^.

Even with the assisted reproductive techniques (ARTs) currently available in reproductive medicine, it remains challenging to counteract the decline in fertility linked to maternal age, particularly beyond the age of 35 years^1^. Nevertheless, in 1% of women, premature ovarian failure (POF) occurs, characterized by disrupted menstrual cycles, elevated serum levels of follicle-stimulating hormone, and diminished anti-Müllerian hormone prior to the age of 40 years. POF can arise from various factors, including genetic predisposition, environmental influences, infections, autoimmune disorders, as well as chemotherapy and ovarian surgery, exerting a significant impact on fertility even among younger women^4^.

With the goal of increasing the chances of pregnancy for women with diminished ovarian reserve or even those experiencing POF, the use of oocytes donated by younger donors for women with reduced ovarian reserve or POF began in the 1980s. Consequently, donated oocytes (DO) have emerged as a viable alternative to autologous oocyte use in ART, yielding good live birth rates in this group^5^. The pregnancy rate among women who receive DO remains more stable, even with advanced maternal age, *compared to women who use their own oocytes*, where the decline in live birth rate with age is much greater^6^. Overall, the live birth rate with DO is equivalent to 19.3% of all live births with ART^7^.

In pregnancies involving DO, the fetus is completely allogeneic, that is, it has a completely distinct genetic composition from the mother who received the oocyte. This is because the fetus inherits genes from both the father and the oocyte donor. Conversely, in spontaneous pregnancies or pregnancies with the mother's own oocyte, the fetus is semi-allogenic to the pregnant woman. During pregnancies with DO, the maternal immune system requires additional adaptations to support the allogeneic nature of the pregnancy^8^. It is believed that the genetic condition of the fetus conceived through DO may trigger immunological responses and placental alterations in the recipient^9^. These alterations can affect the presence and functionality of macrophages, depending on the type of pregnancy, whether spontaneous, ART with or without DO^10^.

There are several reports comparing pregnancies achieved through ART using autologous oocytes versus those using DO. It appears that DO is independently associated with high rates of placental abnormalities, such as pre-eclampsia^11^. When DO was added to the ART, the risk of pre-eclampsia ranged from 4 to 7.94 times higher than in spontaneous pregnancies^12,13^.

Previous research on the obstetric and perinatal risks associated with DO in Brazilian patients was lacking. Conducting a study of these risks within Brazilian patients is imperative given the increasing importance and use of this technique in reproductive medicine in Brazil. Identifying and evaluating these risks is crucial for improving regulations and clinical guidelines related to DO. Our hypothesis is that Brazilian patients who have used DO have a higher risk of adverse perinatal outcomes compared to the group of women who use their own oocytes in ICSI cycles.

The aim of this study was to compare the obstetric and perinatal complications of women who became pregnant with autologous oocytes with those who received DO in ICSI cycles.

## METHODS

A retrospective cohort study was carried out by collecting data from patients at the Profertil Reproductive Medicine Clinic in Niterói, RJ, southeast of Brazil from medical records between 2019 and 2022. This study was approved by the Ethic Committee of Fluminense Federal University (CAAE: 65041422.8.0000.5243).

Only patients who underwent ICSI in an induced cycle using their own or freshly DO, with male infertility factor and tubal factor, were included. Pregnant women with twin pregnancies, regardless of the origin of the oocyte, and pregnant women with pre-existing comorbidities that could negatively impact pregnancy (such as diabetes mellitus, chronic hypertension, and benign or malignant degenerative diseases) were excluded.

A total of 120 patients were evaluated, comprising 51 cases with own oocytes (control group) and 69 cases with DO (study group). Obstetric information (gestational age, type of delivery, presence of diseases during pregnancy, placental abnormalities, and obstetric complications) and perinatal information [birth weight and neonatal intensive care unit ICU admission] were obtained.

The data were transferred to an Excel 2007 spreadsheet (Microsoft Corp., Redmond, WA, USA) and analyzed using SPSS version 20.0 (SPSS Inc., Chicago, IL, USA) and Prisma GraphPad version 7.0 (GraphPad Software, San Diego, CA, USA). Shapiro-Wilk normality tests were used to analyze whether the values were Gaussian distributed. Parametrically distributed values were presented as medians and standard error (SE) or standard deviation (SD). To carry out the analyses, the following variables were initially considered dependent variables: age, ethnicity, type of delivery, gestational age at delivery, race, obstetric and perinatal complications, birth weight, and admission to neonatal and maternal ICU.

Given that we did not observe a normal distribution of ages between the groups, the age factor was then included in the model as an independent variable due to its physiological importance. Women under 40 years of age use their own oocytes more often and women over 40 years of age use DO. Therefore, treatments (own and DO) and age (<40 years and ≥40 years) were considered independent variables. Two-way ANOVA (with Tukey's post-test) and the Kruskal-Wallis test (with Dunn's post-test) were carried out to compare dependent variables between the treatment groups and age. Categorical variables were described as absolute and percentage frequencies and are presented in tables. Binary logistic regression was used to calculate the odds ratio (OR) for adverse pregnancy and neonatal outcomes. Stepwise binary logistic regression with maternal age as a covariate was used to adjust the model and calculate the adjusted OR. The level of significance for all tests was p<0.05.

## RESULTS

From April 2019 to September 2022, 120 patients undergoing ICSI were evaluated. The final statistical analysis included 51 cases of own oocytes (control group) and 69 cases of DO (study group).

Patients who received DO were significantly older than those who received their own oocytes (41.96±2.16 vs 38.54±1.42 years, p<0.001). There was no significant association between the origin of the oocyte and gestational age at delivery (p=0.296) or birth weight (p=0.836). There was also no significant association between the origin of the oocyte and white (p=0.119), mixed (p=0.818), Black (p=0.071), vaginal delivery (p=0.399), cesarean section (p=0.399), presence of obstetric and perinatal complications (p=0.366), admission to neonatal ICU (p=0.120), and maternal admission to adult ICU (p=0.767) (Table 1).

**Table 1:** Maternal characteristics and perinatal outcomes of both included groups.

		Donated oocytes (N=69)	Own oocytes (N=51)	p
Age (years ± SD)		41.96 ± 2.16	38.54 ± 1.42	<0.001¥
Ethnicity				
	White	59.4% (41/69)	74.5% (38/51)	0.119∂
	Mixed	20.3% (14/69)	17.6% (9/51)	0.816∂
	Black	20.3% (14/69)	7.8% (4/51)	0.072∂
Type of delivery				
	Vaginal	2.9% (2/69)	7.8% (4/51)	0.399∂
	Cesarean section	97.1% (67/69)	92.2% (47/51)	0.399∂
	Gestational age at delivery (weeks±SE)	37.8±0.38	37.69 ± 0.35	0.296¥
	Obstetric and perinatal complications	43.5% (30/69)	35.3% (18/51)	0.366∂
	Birth weight (g±SE)	3182.68±98.73	3036.43 ± 92.33	0.836¥
	Admission to neonatal ICU	8.7% (6/69)	2.0% (1/51)	0.120∂
	Maternal ICU admission	7.2% (5/69)	5.9% (3/51)	0.767∂

ICU: intensive care unit; SE: standard error; SD: standard deviation. Mann-Whitney: median (minimum-maximum)

¥ Chi-squared: % (n/N)

∂ p<0.05.

The origin of the oocyte did not show association and no significant increase in the risk of pre-eclampsia (p=0.357), gestational diabetes mellitus (p=0.187), premature rupture of ovular membranes (p=0. 996), uterine atony (p=0.996), placenta previa (p=0.393), oligohydramnios (p=0.393), and gestational hypertension (p=0.393), even after maternal age was included as a covariate in the model (Table 2).

**Table 2:** Association between oocyte type and obstetric and perinatal complications.

Obstetric and perinatal complications	Donated oocytes (N=69)	Own oocytes (N=51)	OR (95%CI)	aOR (95%CI)
Preeclampsia	17.4% (12/69)	15.5% (8/51)	1.32 (0.42–3.06), p>0.999	1.74 (0.53–5.65), p=0.357
Gestational diabetes mellitus	10.1% (7/69)	3.9% (2/51)	2.76 (0.56–13.55), p=0.298	3.51 (0.54–22.69), p=0.187
Premature rupture of ovular membranes	1.4% (1/69)	2.0% (1/51)	0.73 (0.03–14.23), p>0.999	0.00 (0.0-infinite), p=0.996
Uterine atony	1.4% (1/69)	2.0% (1/51)	0.73 (0.03–14.23), p>0.999	0.00 (0.0-infinite), p=0.996
Placenta previa	1.4% (1/69)	3.9% (2/51)	0.36 (0.02–3.19), p=0.574	0.22 (0.008–6.72), p=0.393
Breech presentation	1.4% (1/69)	0.0% (0/51)	*	
Polycystic kidneys	1.4% (1/69)	0.0% (0/51)	*	
Oligohydramnios	1.4% (1/69)	3.9% (2/51)	0.36 (0.02–3.19), p=0.574	0.22 (0.008–6.72), p=0.393
Gestational hypertension	1.4% (1/69)	3.9% (2/51)	0.36 (0.02–3.19), p=0.574	0.22 (0.008–6.72), p=0.393
Eclampsia	1.4% (1/69)	0.0% (0/51)	*	
HELLP syndrome	0.0% (0/69)	0.0% (0/51)	*	
Polyhydramnios	1.4% (1/69)	0.0% (0/51)	*	
Chorioamnionitis	1.4% (1/69)	0.0% (0/51)	*	
Respiratory distress	1.4% (1/69)	0.0% (0/51)	*	

Binary logistic regression; OR: odds ratio; aOR: odds ratio adjusted for maternal age; CI: confidence interval; Chi-square: % (n/N);

* It was not possible to use a statistical test due to the absence of at least one case in each group. p<0.05.

## DISCUSSION

This study examined obstetric and perinatal outcomes among Brazilian pregnant women who underwent ICSI using either autologous or DO. In contrast to most studies, an increase in obstetric and perinatal complications was not observed in pregnancies with DO compared to pregnancies with autologous oocytes.

DO is a widely employed ART technique, particularly in cases of female infertility related to oocyte quality due to advanced maternal age, POF, early menopause, or even treatments or medical conditions affecting ovarian reserve, such as chemotherapy and genetic disorders. Currently, DO is utilized in at least 7% of all in vitro fertilization (IVF/ICSI) cycles in Europe. However, this number of cycles is likely to be higher, as not all countries report their DO data^8^. DO from younger donors exhibit a significantly higher IVF/ICSI success rate compared to autologous oocytes from women over 40 years of age. This underscores the critical role of oocyte quality in successful fertilization and subsequent embryo development^2,7^.

In this study, 120 patients were evaluated, 51 cases of own oocytes (control group) and 69 cases of DO (study group). The mean maternal age was higher in the DO group (41.96 years) than in the control group (38.54 years). While only 33.3% of the controls were over 40 years of age, 82.6% of the DO group were over 40 years of age. As has been reported in the literature, DO patients are usually older than those who use their own oocytes, as the procedure is indicated for cases of advanced maternal age^8,14^.

Studies assessing the impact of donor ethnicity have indicated that Black race appears as a risk factor for unsuccessful pregnancy outcomes following DO, in contrast to the higher likelihood of pregnancy observed in White recipient women. According to Bodri et al.^15^, the explanation for this is that Black women have more tubal infertility, uterine myomas, and a higher body mass index than other races. In addition, a retrospective study by Liu et al.^16^ described that the Black recipient who received an oocyte from a Black donor appeared to have a lower likelihood of achieving a live birth compared to White donor–recipient pair. In this study, both the DO and control groups were ethnically homogeneous, with no statistically significant difference observed. Furthermore, the absence of Black recipients who did not conceive or did not carry the pregnancy to term with a live birth might explain the lack of racial disparity between the groups.

There are several reports on the prevalence of cesarean section in cases of DO compared to pregnancies with autologous oocytes, showing at least twice the risk of occurrence, regardless of whether the embryo transfer was fresh or frozen. While the cesarean section rate in our study ranged from 92 to 97%, it is notably higher than the average rate reported in the literature, which stands at around 45%^17,18^. The exception was a German study in which the frequency of cesarean section in the group of women with oocyte receptors in a singleton pregnancy was 83.9%^19^. The high cesarean section rates observed in our study are consistent with the high rates observed in Brazil, especially in private services^20^.

In our study, despite observing a higher numerical frequency of preterm births in women with DO, no statistically significant relationship was found between preterm births and oocyte origin. In the study by Elenis et al.^21^, there was also no statistically significant relationship between prematurity and DO, with only a trend toward prematurity in the DO group.

In this study, pregnant women>40 years of age had more obstetric complications compared to pregnant women<40 years of age, and DO patients also exhibited a higher incidence of obstetric complications compared to pregnant women with their own oocytes. However, no statistically significant difference was observed, possibly due to the limited number of patients evaluated. A higher incidence of obstetric and perinatal complications among patients receiving DO has been previously described in the literature^17,18^. Obstetric complications are known to be more frequent in older pregnant women^14,22^, particularly when DO is performed after the age of 45 years^14^.

In a meta-analysis of 23 studies, DO was associated with a higher risk of hypertensive disorders of pregnancy, pre-eclampsia, severe pre-eclampsia, and pregnancy-induced hypertension. The meta-analysis also found a 1.27 times higher risk of gestational diabetes mellitus in DO patients^5^. In a retrospective cohort study that compared pregnant women from DO (n=78) and pregnant women with own oocytes (n=112), the authors observed that pregnancy-induced hypertension and gestational diabetes mellitus were significantly higher in DO patients^23^.

In this study, the newborns of DO patients had a relatively higher mean birth weight than the control group; however, the difference was not significant. In the study of Rodriguez-Wallberg et al.^24^, as in our study, no difference in birth weight was observed between DO and ART patients. A meta-analysis of 23 studies also found no increased risk of low birth weight when adjusting for the presence of pre-eclampsia^5^.

Obstetric and perinatal complications are known to increase the likelihood of maternal and neonatal ICU use during the postpartum period. In this study, DO patients had a numerically higher rate of ICU admission for both neonates and mothers, although there was no statistical significance. Malchau et al.^25^ compared patients who underwent DO with IVF or ICSI and spontaneous pregnancy and found a statistically significant difference in terms of longer ICU stay in DO patients compared to patients who underwent ICSI with their own oocyte or spontaneous pregnancy. On the contrary, patients who underwent IVF with their own oocytes had the same length of stay as DO patients.

A limitation of this study was the sample size, particularly in the control group, attributed to the difficulty in recruiting patients over the age of 40 years who utilized their own oocytes in an ICSI procedure. Nevertheless, this study marks the first exploration in Brazil of obstetric and perinatal data among patients who underwent DO.

Thus, according to our data, DO can be considered a safe alternative for patients with low ovarian reserve due to advanced age or other factors. However, it is important to note that there may be a higher risk of DO in certain aspects, especially in patients over the age of 40 years, with pre-existing chronic diseases and with poor access to prenatal care. Thus, obstetric follow-up needs to be individualized with strategies to reduce the risk of complications, with special attention to hypertensive disorders during pregnancy.

## CONCLUSION

No increase in obstetric and perinatal complications was observed in DO pregnancies compared with autologous oocyte pregnancies in women undergoing ICSI without prior comorbidities. Further studies with larger sample sizes are needed to validate our findings.
